# A quantitative analysis method for comitant exotropia using video-oculography with alternate cover

**DOI:** 10.1186/s12886-018-0747-9

**Published:** 2018-03-22

**Authors:** Nohae Park, Byunggun Park, Minkyung Oh, Sunghyuk Moon, Myungmi Kim

**Affiliations:** 1 Department of Ophthalmology, Busan Paik Hospital, Inje University College of Medicine, 75 Bokji-ro, Busanjin-gu, Busan, 47392 Republic of Korea; 2 Department of Pharmacology, Busan Paik Hospital, Inje University College of medicine, Busan, Republic of Korea; 3 Department of Ophthalmology, Yeungnam University College of Medicine, Daegu, Republic of Korea

**Keywords:** Strabismus, Video-oculography, Ocular deviation, Alternate cover

## Abstract

**Background:**

The purpose of this study was to evaluate the efficacy of a quantitative analysis method for comitant exotropia using video-oculography (VOG) with alternate cover.

**Methods:**

Thirty-four subjects with comitant exotropia were included. Two independent ophthalmologists measured the angle of ocular deviation using the alternate prism cover test (APCT). The video files and data of changes in ocular deviation during the alternate cover test were obtained using VOG. To verify the accuracy of VOG, the value obtained using VOG and the angle of a rotating model eye were compared, and a new linear equation was subsequently derived using these data. The calculated values obtained using VOG were compared with those obtained using the APCT.

**Results:**

Rotation of the model eye and the values obtained using VOG demonstrated excellent positive correlation (*R* = 1.000; *p* < 0.001). A simple linear regression model was obtained: rotation of the model eye = 0.978 × value obtained using VOG for a model eye – 0.549. The 95% limit of agreement for inter-observer variability was ±4.63 prism diopters (PD) for APCT and that for test-retest variability was ±3.56 PD for the VOG test. The results of APCT and calculated VOG test demonstrated a strong positive correlation. Bland-Altman plots revealed no overall tendency for the calculated values obtained from VOG to differ from those obtained using APCT.

**Conclusions:**

VOG with alternate cover is a non-invasive and accurate tool for quantitatively measuring and recording ocular deviation. In particular, it is independent of the proficiency of the examiner and, can therefore, be useful in the absence of skilled personnel.

**Trial registration:**

ClinicalTrial.gov, NCT03119311, Date of registration: 04/17/2017, Date of enrolment of the first participant to the trial: 04/25/2017.

## Background

Accurate measurements are important for planning strabismus surgery. Methods available for measuring the angle of ocular deviation include the alternate prism cover test (APCT), Hirschberg test, and Krimsky test. In APCT, the subject gazes at the target with both eyes, a prism is placed in front of the uncovered eye, and an alternate cover test is performed. The angle is measured by increasing or decreasing the strength of the prism used until there is no deviation or the deviation is reversed. However, the prism must be changed several times, especially when there is an accompanying vertical strabismus. Consequently, the duration of the examination will be long in such cases and, as such, it is difficult to perform in a child who does not cooperate or gaze in accordance with instructions. In such situations, the Hirschberg and Krimsky tests are used to determine the angle of deviation. The Hirschberg test measures the distance between the corneal light reflex and the center of the pupil, and then converts it into an angle. Although it is a relatively simple method, it may not accurately measure the exact ocular deviation. The Krimsky test measures the angle of ocular deviation using a prism and the corneal light reflex. For this reason, APCT measures the entire deviation, including tropic and phoric components, whereas the Krimsky tests only measure the tropic component. Both the Hirschberg and Krimsky tests require correction of angle kappa, which is largely subjective and, therefore, can lead to inter-observer errors. When the goal of surgery is to achieve orthotropia, APCT should be used whenever possible [1]. In addition, such tests may have limitations in recording eye movements themselves.

On the other hand, a scleral search coil can be used to objectively record and measure ocular deviation. This is an accurate method because it has a spatiotemporal resolution < 1° and < 1 ms. However, it is difficult to wear a scleral search coil for more than 30 min because it is worn on the limbus of the cornea. Therefore, photography and video-oculography (VOG) have been proposed as methods to measure eye movements noninvasively and objectively [2–6]. VOG has demonstrated a measurement error < 1° for an eye movement range < 40°, and a high correlation (R^2^ = 0.99) with the scleral search coil method for both horizontal and vertical eye movements below 15° [6, 7]. In particular, several studies have reported measuring ocular deviation in intermittent exotropia using VOG [8–10]. To our knowledge, there has been no attempt to quantitatively measure ocular deviation using VOG with dissociation of both eyes. We believe it is important to measure ocular deviation with dissociation of both eyes, similar to APCT, for surgery. Therefore, we attempted a method of using VOG with alternate cover for non-invasive and reliable measurement of the angle of ocular deviation. Additionally, we evaluated VOG as an alternative method to standard tests to determine whether it can obviate these limitations.

## Methods

This prospective study was performed at the Department of Ophthalmology, Inje University Busan Paik Hospital. All aspects of the research protocol complied with the tenets of the Declarations of Helsinki and were approved by the Institutional Review Board of Inje University Busan Paik Hospital (Busan, Korea). Written informed consent was obtained from all parents or legal guardians; children and adolescent assent forms were also provided for children 7 years of age and older.

### Calibration of VOG using a model eye

**Fig. 1:**
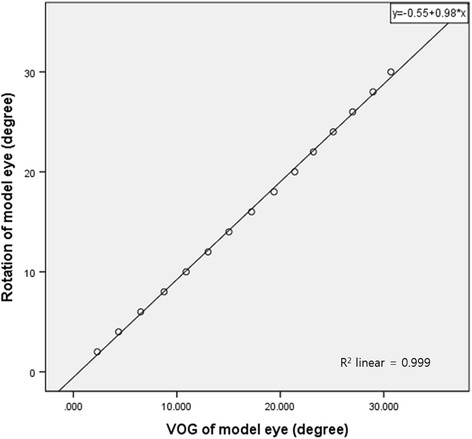
The rotation of the model eye and the value obtained using video-oculography demonstrated excellent positive correlation (*R* = 1.000; *p* < 0.001) (rotation of model eye [degrees] = 0.978 × value obtained from video-oculography of the model eye [degrees] – 0.549; R^2^ = 0.999)

A model eye with a pupil diameter of 5.5 mm and globe diameter of 26 mm was used to verify the accuracy of VOG. A protractor was attached to the center of the eyeball to verify the amount of rotation of the eyeball. Each of the two experiments was repeated four times, and a total of eight experiments were performed. The eyeball was rotated from 0° to 30° at intervals of 2°. The video taken of the model eye stopped for > 5 s every 2°. The degree of rotation of the eyeball and the values obtained using VOG were compared. Based on the results of the analysis, a linear regression equation for the degree of eyeball rotation was derived. In the reliability analysis of eight repeated VOG tests for a model eye, the intra-class correlation coefficient was 1.000 (95% confidence interval [CI] 1.000 to 1.000; *p* < 0.001). The intra-class correlation coefficient between the first measurement of two independent examiners was 1.000 (95% CI 0.999 to 1.000; *p* < 0.001). The VOG demonstrated excellent agreement among all eight repeated examinations. The linear regression equation was derived from the mean value of the eight VOGs and the rotated angle of the model eye using linear regression analysis (Fig. 1).

$$ \mathrm{Rotation}\ \mathrm{angle}\ \mathrm{of}\ \mathrm{the}\ \mathrm{model}\ \mathrm{eye}\ \left(\mathrm{degrees}\right)=0.978\times \mathrm{angle}\ \mathrm{obtained}\ \mathrm{using}\ \mathrm{VOG}\ \left(\mathrm{degrees}\right)\hbox{--} 0.549 $$

$$ \mathrm{Calculated}\ \mathrm{VOG}\ \mathrm{value}\ \left(\mathrm{prism}\ \mathrm{diopters}\right)=\tan\ \left(\mathrm{rotation}\ \mathrm{angle}\ \mathrm{of}\ \mathrm{the}\ \mathrm{model}\ \mathrm{eye}\ \left[\mathrm{degrees}\right]\right)\times 100 $$

### Participants

Thirty-four subjects with comitant exotropia who could be observed > 3 times, with a difference between distant and near deviation angle < 3 prism diopters (PD), were enrolled. All subjects underwent APCT to measure the angle of ocular deviation. On the same day, VOG (SLMED, Seoul, Korea) was performed and APCT was performed 30 min later. Subjects with incomitant strabismus, horizontal deviation > 50 PD in APCT, ocular comorbidity other than strabismus or with systemic disease, refractive errors > 6.00 diopters, those not willing to undergo VOG, children < 4 years of age, and subjects wearing spectacles during measurements were excluded.

### Comparison of APCT and VOG

Two independent ophthalmologists performed APCT using a plastic prism set (Luneau SAS, Prunay LeGillon, France). The subjects were asked to fixate on a black-on-white optotype at 3 m, which subtended a visual angle of 50 min of arc (MOA), equating to a Snellen optotype of 20/200.

**Fig. 2:**
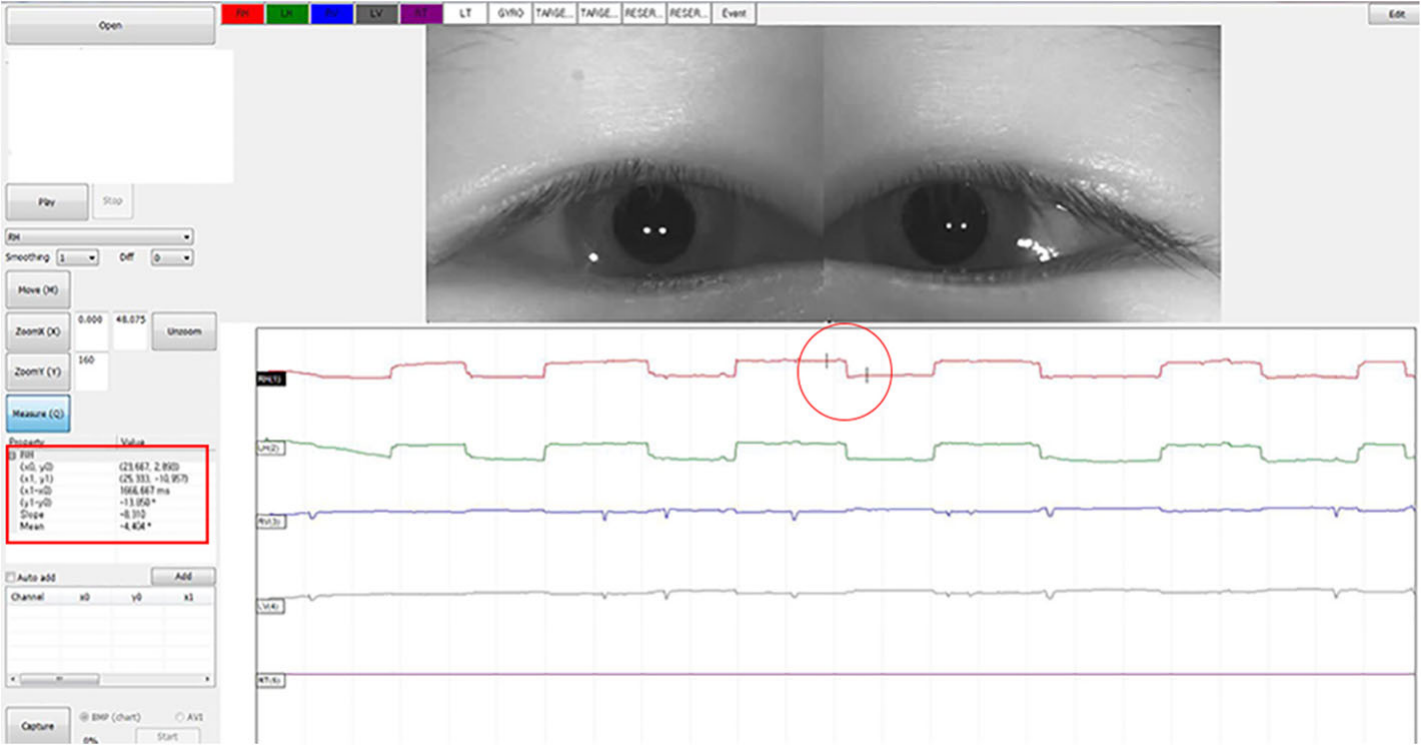
Interface of the video-oculography device. This image reveals the degree of movement of the eyeball in the form of a graph. The red line refers to the movement of the right eye, and the amount of change in the two points (in the circle) is represented by an angle in the square

The VOG equipment used in the present study had a tilted semi-transparent glass through which the subject could gaze at the target with a red light with a visual angle of 50 MOA in the monitor situated at 1 m. The subject wearing the VOG goggles was instructed to look at the red light, between the two eyes, with head position kept straight so that during the examination the eyes were in primary position. During the first 10 s, initial binocular alignment was verified with both eyes open. Subsequently, each eye was allowed 5 s of covered time and 5 s of uncovered time, and the alternate cover test was repeated 5 times, with each eye being covered for 5 s. A 120 Hz camera was used for VOG, and the eyeball was observed to deviate during the alternate cover test; the magnitude of deviation was subsequently obtained (Fig. 2). The obtained values ​​were expressed in degrees (°), and were substituted into the linear regression equation derived earlier using the model eye. The new angle values thus obtained were converted to PD to compare with the values obtained using APCT.

### Main outcome measure

The inter-observer variability of APCT performed by two independent examiners and the inter-visit variability of four examinations by one examiner were measured. The VOG recorded the distance moved by the eye during re-fixation after its deviation in the alternate cover; three repeated test values ​​were obtained and a reliability analysis was performed. The first value among the results obtained using VOG was used in the linear regression equation and subsequently compared with the value obtained using APCT.

### Statistical analysis

SPSS version 18.0 (SPSS, Inc., Chicago, Illinois, USA) was used for statistical analysis. The Pearson correlation coefficient was used to determine the linear relationship between VOG and the model eye, and the linear regression equation was derived using linear regression analysis. The reliabilities of APCT and VOG were evaluated using intra-class correlation coefficient, and consistent variability between APCT and VOG was represented using a Bland-Altman plot. Correlation between the two tests was calculated using the Pearson correlation coefficient.

## Results

### Participants

Thirty-four subjects with comitant exotropia, of whom 22 (64.7%) were female, were included in the present study. The mean age was 13.7 ± 11.2 years (range, 5–51 years). Thirty-one of 34 subjects had uncorrected visual acuity better than 20/30 in both eyes; uncorrected visual acuity in either eye of the other three subjects was not worse than 20/70. APCT and VOG were performed in all subjects (Table 1).

**Table 1:** Mean angle of exotropia measured using alternate prism cover test and video-oculography

Value	Alternate prism cover test (*n* = 34)		Video-oculography (*n* = 34)	
	Examiner 1	Examiner 2	Average value of three test-retests	Calculated value from linear equation
Degrees	14.32 ± 3.66 (6.84–23.27)	13.84 ± 4.14 (5.71–24.23)	15.47 ± 3.91 (8.50–26.58)	14.58 ± 3.83 (7.77–25.44)
Prism diopters	25.65 ± 6.88 (12–43)	24.79 ± 7.81 (10–45)	27.81 ± 7.52 (14.95–50.02)	26.13 ± 7.28 (13.64–47.57)

Data presented as mean ± SD (range)

### Comparison of APCT and VOG

**Fig. 3:**
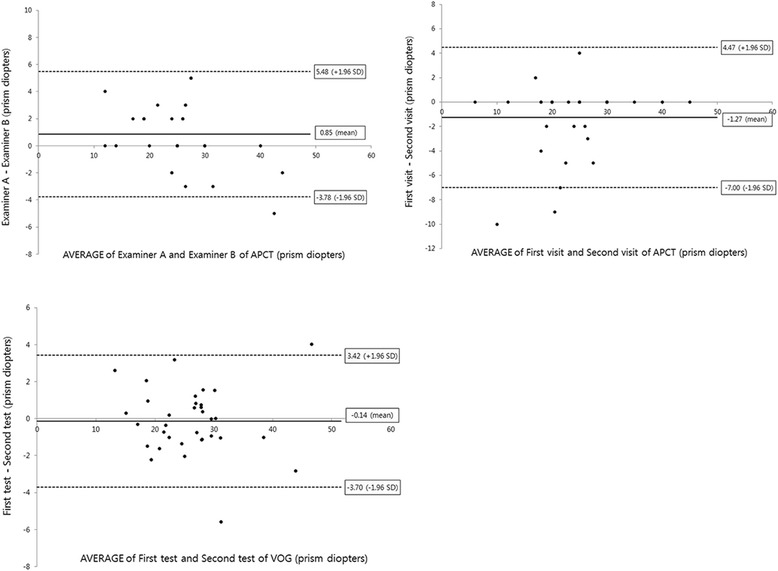
Bland-Altman plots revealing interobserver variability and inter-visit variability for the alternate prism cover test (APCT) and test-retest variability for video-oculography with alternate cover. Upper and lower dotted lines represent the 95% limits of agreement. The half-width of the 95% limit of agreement measured of the interobserver variability (top left), inter-visit variability (top right) for APCT and test-retest variability (bottom) for video-oculography were 4.63 prism diopters (PD), 5.74 PD and 3.56 PD, respectively

The inter-observer variability for APCT was determined using the results from two independent examiners. The Bland-Altman plot demonstrated consistent variability, except for one subject with deviation > 40 PD. The half-width of the 95% limit of agreement was ±4.63 PD (Fig. 3 [top left]). On reliability analysis, the inter-observer correlation coefficient was 0.974 (95% CI 0.947 to 0.987; *p* < 0.001).

The inter-visit reliability of the APCT was determined for 24 of 34 subjects who had been examined by one examiner > 4 times in three months; the inter-visit correlation coefficient was 0.968 (95% CI 0.941 to 0.985; *p* < 0.001). The Bland-Altman plot between the first and second APCT demonstrated consistent variability, except for two subjects, the half-width of the 95% limit of agreement being ±5.74 PD (Fig. 3 [top right]).

Thirty-four subjects were included in the VOG test. Reliability analysis of the three VOG readings demonstrated high agreement (0.990 [95% CI 0.983 to 0.995]; *p* < 0.001), and the half-width of the 95% limit of agreement on Bland-Altman plot was ±3.56 PD (Fig. 3 [bottom]).

Of the 34 subjects, 28 (82.4%) exhibited a difference in ocular deviation of < 3 PD between VOG and APCT, and 32 (94.1%) demonstrated a difference of < 5 PD (Table 2).

**Table 2:** Differences between alternate prism cover test and video-oculography

Prism diopters	Subjects, n (%)
< 3	28 (82.4)
3–5	4 (11.7)
> 5	2 (5.9)
Total	34 (100)

**Fig. 4:**
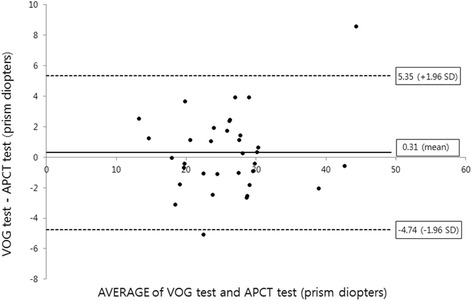
Bland-Altman plot comparing the values obtained using the alternate prism cover test (APCT) and video-oculography (VOG) test. The Bland-Altman plot demonstrated consistent variability. The half-width of the 95% limit of agreement was ±5.05 prism diopters. There was no overall tendency for the values obtained using VOG to differ from those obtained using APCT

**Fig. 5:**
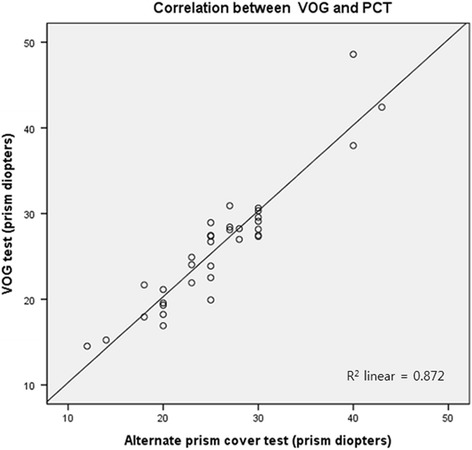
Scatter plot and Pearson correlation between values calculated using video-oculography (VOG) and the values obtained using the alternate prism cover test (APCT). The values calculated from VOG demonstrated a strong correlation with the values obtained using APCT (*R* = 0.934; *p* < 0.001)

The Bland-Altman plot of VOG and APCT demonstrated consistent variability, except for two subjects, the half-width of the 95% limit of agreement being ±5.05 PD (Fig. 4). Furthermore, there was also a strong positive linear relationship between the two tests (*R* = 0.934; *p* < 0.001) (Fig. 5).

## Discussion

The purpose of our study was to measure the objective angle of ocular deviation using VOG with alternate cover in subjects with exotropia. The principle of the VOG device used in our study was that light was transmitted through the tilted semi-transparent glass and the subject could look at the target. As the light was being reflected, the two cameras could record the movement of the eyes without blocking the visual axes. The pupil was detected in real time, and the deviation of the eyeball was assessed by measuring the change in the reference point of the center of the pupil.

Although APCT may represent a typical test for measuring angles of deviation, the results can differ because of differences in measurements using prisms made by individual examiners. Therefore, error involved in the measurements are dependent on the skill of the examiner and the cooperation of the subject [11–13]. In particular, the Pediatric Eye Disease Investigator group [11] suggested that two skilled observers may have an error ≥ 12 PD in the measurement of esotropia exceeding 20 PD, and an error ≥ 6 PD in the measurement of 10 to 20 PD esotropia. The angle of ocular deviation measured by one observer demonstrated that the 95% limit of agreement on a measurement was ±7.3 PD for esotropia exceeding 20 PD, and ± 4.1 PD for 10–20 PD esotropia at distance. In addition, APCT could not record eye movement itself; therefore, it depends on the record of the examiner. For this reason, methods using photography were used for measurements that are more objective. Among them, Yang et al. [2] took pictures at a distance of 1 m, and the corneal light reflex points and limbus locations were extracted from two-dimensional photographs and analyzed using a three-dimensional strabismus photo analyzer (R & DB Foundation, Seoul, Republic of Korea). The results demonstrated high correlation with the Krimsky test. This is useful to examine cases of manifest strabismus. However, because this test did not dissociate the two eyes, the angle of ocular deviation would be variable according fusion of both eyes in intermittent exotropia, or changeable depending on dominant eye in incomitant strabismus. Therefore, there are restrictions to its use in intermittent exotropia and incomitant strabismus. Additionally, there are limitations in measuring the angle of ocular deviation for retinopathy of prematurity with macular dragging because the angle kappa cannot be considered. In another improvement study, the use of an infrared ray filter and an infrared camera with this method was proposed to observe the deviation angle in patients with latent strabismus [3].

An alternative for objectively measuring eye movements is to use a VOG device equipped with a camera at a sample rate of 200 to 250 Hz. In previous studies, it was reported to have high correlation with the scleral search coil in the fixation position [6, 7]. The VOG device used in our study had a frequency of 150 Hz; hence, it was less accurate than the scleral search coil used to assess rapid eye movements. However, because our study was intended to measure the distance moved during an eyeball deviation, the results were not greatly influenced by camera frequency. In our study, we needed a device verification step to accurately measure the amount of eyeball rotation. The results obtained using VOG and the amount of eyeball rotation showed statistically high correlation (linear correlation coefficient = 1.000; *p* < 0.001), and a linear regression equation of the eyeball rotation angle with the angle obtained using VOG was derived. Using this equation, the degree of ocular change in the subject group measured using VOG was converted to actual eyeball rotation angle values. These converted values ​​were compared with the angles of ocular deviation obtained using APCT. The Bland-Altman plot between the APCT values and the calculated VOG values showed consistent variability, except for two subjects. As for the outliers, one subject exhibited ocular deviation values of 20, 20, and 25 PD from the three APCTs, and 19.92 PD from VOG testing. In our study setting, ocular deviation was analyzed based on the last visit, and was judged to be located outside the 95% limits of agreement. The other subject had exotropia with inferior oblique overaction in both eyes. It was V-pattern exotropia with 55 PD in the upward gaze, 40 PD in the primary position, and 35 PD in the downward gaze. We presume that the upward gaze may have been the cause of the positive difference despite maintenance of head position.

The advantage of using VOG is that all eye movements can be recorded as video recordings, and the eye tracking system can record both eye movements and the angle of ocular deviation. Another advantage is the dissociation of the two eyes using alternate covering, and measurement of the distance moved by the eye by recording a video. Hence, VOG is not influenced by the angle kappa. Several studies reporting the objective measurement of strabismus were influenced by the angle kappa because their assessments were based on corneal light reflex points. In addition, the measurement of the manifest strabismus alone was performed without dissociating the two eyes [2, 5, 14–16]. In contrast, in a study measuring the angle of ocular deviation by dissociating the two eyes, Yang et al. [3] used an infrared transmission filter for dissociation and measured the ocular deviation using photographs. However, because photographs―unlike video recordings―do not reflect the continuity of time, it is different from the method used in our study in that it does not record eye movement in real time. Therefore, if we analyze the angle of ocular deviation by the method used in our study with the aid of VOG, we can analyze deviation patterns in dissociated strabismus cases and use it for screening in intermittent exotropia with good convergence. Second, it is also useful for measuring the maximum angle of ocular deviation, which is significant in intermittent exotropia. Third, it can measure the ocular deviation in a short time. It took approximately 2 min during the VOG test (1 min for wearing goggles, 1 min for performing VOG with alternate cover). Finally, because VOG can quantitatively record and compare eye movements in both eyes separately, it is possible to accurately analyze the difference between secondary and primary deviations in incomitant strabismus. This may be useful for follow-up observations. The purpose of our study was to evaluate the accuracy of VOG and to include the inter-visit variability of a single examiner in the process of comparing and analyzing the degree of variability of APCT measurements. However, paralysis, a type of incomitant strabismus, was excluded from our study because there can be changes in ocular deviation during recovery. In the future, we will perform a comparative analysis of both eyes in cases of incomitant strabismus such as paralysis.

The first limitation of our study was that children < 4 years of age were excluded from the evaluation of the accuracy of VOG. We suspect that younger children have a lower attention span and ability to fixate well. Moreover, subjects with claustrophobia could not be assessed because the video goggles had to be worn at the time of the test. Second, subjects did not wear glasses, even if there was a refractive error, although the author found that the VOG goggle supported the use of glasses and video camera could detect the center of the pupil beyond the glasses, the results were affected by the prism effect of glasses. The aim of our study was to evaluate a method using VOG with alternate cover to measure ocular deviation in lieu of standard tests. Therefore, it was important to demonstrate the accuracy of VOG. Subjects did not wear glasses to eliminate variables caused by the prism effect. Third, the target was a red light (50 MOA) for the VOG test and a black-on-white optotype (50 MOA) for the APCT. For measuring the angle of ocular deviation, we did not use a light source as the target; instead, the visual acuity chart was used because the accommodation levels are different. If a light is used as the target, the accommodation is less than that with the acuity chart; hence, the deviation will be lesser for esotropia and vice versa for exotropia [4]. The VOG goggle is constructed from semi-transparent glass, which can degrade contrast of letter targets. The same a black-on-white optotype in VOG and APCT may be different. Surmising that red light would better attract subject attention, it was used instead of a black-on-white optotype for evaluating VOG. However, no difference between red light and a black-on-white optotype was observed. A possible reason is that subjects may perceive the red light as a red dot with higher resolution through the semi-transparent glass. In addition, a recent study using a prism cover test for far distance in intermittent exotropia reported no significant variability between light and a black-on-white optotype target [17], supporting our trial to use red light for the VOG test. Finally, the distance of the fixation target in VOG was different from that in APCT for measuring ocular deviation because the distance was set up by a meter in the software of VOG test. For reducing the variability caused the distance, we limited our study to a group of subjects with < 3 PD difference between near and far distance ocular deviation.

## Conclusion

VOG with alternate cover can be used to quantitatively and non-invasively measure the angle of ocular deviation, and more reliably. Moreover, the corrected values demonstrate high agreement with APCT values. Because this test is not significantly influenced by the skill of the examiner, it can accurately measure the angle of ocular deviation, even in the absence of an expert and record of ocular deviation itself. In particular, it is highly valuable as a screening test that can detect strabismus in many patient populations.

## Abbreviations

APCT: Alternate prism cover test
MOA: Min of arc
PD: Prism diopters
VOG: Video-oculography
